# Features of Hemophagocytic Lymphohistiocytosis in Infants With Severe Combined Immunodeficiency: Our Experience From Chandigarh, North India

**DOI:** 10.3389/fimmu.2022.867753

**Published:** 2022-06-23

**Authors:** Pandiarajan Vignesh, Gummadi Anjani, Rajni Kumrah, Ankita Singh, Sanjib Mondal, Johnson Nameirakpam, Ankur Jindal, Deepti Suri, Madhubala Sharma, Gurjit Kaur, Sathish Sharma, Kirti Gupta, Sreejesh Sreedharanunni, Amit Rawat, Surjit Singh

**Affiliations:** ^1^Allergy Immunology Unit, Department of Pediatrics, Advanced Pediatrics Centre, Postgraduate Institute of Medical Education and Research, Chandigarh, India; ^2^Department of Histopathology, Postgraduate Institute of Medical Education and Research, Chandigarh, India; ^3^Department of Hematology, Postgraduate Institute of Medical Education and Research, Chandigarh, India

**Keywords:** severe combined immunodeficiency, hemophagocytic lymphohistiocytosis, infections, BCG, family history, X-linked

## Abstract

**Background:**

Hemophagocytic lymphohistiocytosis (HLH) is characterized by uncontrolled and excessive inflammation leading to high mortality. Aetiology of HLH can be primarily due to genetic causes or secondarily due to infections or rheumatological illness. However, rarely T-cell deficiencies like severe combined immunodeficiency (SCID) can develop HLH.

**Objective:**

To describe clinical and laboratory features of SCID cases who developed HLH.

**Methods:**

We collected clinical, laboratory, and molecular details of patients with SCID who developed HLH at our center at Chandigarh, North India.

**Results:**

Of the 94 cases with SCID, 6 were noted to have developed HLH-like manifestations. Male-female ratio was 5:1. Median (inter-quartile range) age of onset of clinical symptoms was 4.25 months (2-5 months). Median (inter-quartile range) delay in diagnosis was 1 month (1-3.5 months). Family history of deaths was seen in 4 cases. Molecular defects in *IL2RG* were seen in 5 out of 6 cases. Documented infections include disseminated bacillus calmette-guerin (BCG) infection (n=2), blood stream infections (n=3) with *Staphylococcal aureus* (n=1), *Klebsiella pneumonia* (n=1), and *Pseudomonas aeruginosa* (n=1), pneumonia (influenza H1N1 strain, and K. *pneumoniae* (n=1).

**Conclusion:**

Children with SCID can present with HLH-like manifestations secondary to fulminant infections. A high index of suspicion of SCID is needed in infants who present with HLH who have an associated infection or a suggestive family history. Occurrence of HLH-like manifestations in SCID suggests that T-lymphocytes may not have a significant role in immunopathogenesis of HLH.

## 1 Introduction

Severe combined immune deficiency (SCID) is a heterogeneous group of disorders caused by a variety of genetic abnormalities (1) (2). It is characterized by defective T and B lymphocyte function leading to life threatening infections and mortality if not treated with HSCT on time.

Hemophagocytic lymphohisticytosis (HLH) is a life-threatening condition due to immune dysregulation characterized by multi-organ dysfunction, rapidly progressive cytopenias, hypertriglyceridemia, hypofibrinogenemia, and hyperferritinemia. Genetic causes of primary HLH include *PRF1, STX11, STXBP2, MUNC, UNC13D, RAB27A, LYST, AP3B, SH2D1A*, and *BIRC4* defects or other primary immunodeficiency diseases such as SCID and chronic granulomatous disease (3) (4).

Diagnosis of SCID in an infant presenting with HLH-like manifestations can be a challenge for clinical immunologists in view of symptoms masquerading as sepsis and multiorgan dysfunction. Also, the rapidly progressive bicytopenia/pancytopenia makes interpretation of lymphocyte subsets by flow cytometry a challenging task. Management of HLH-like manifestations in an infant with SCID is equally challenging because almost always, an infection would be the trigger, and treating the infection becomes essential. We report our cohort of 6 patients with SCID from Northern India who developed HLH-like manifestations and provide a brief review of literature. To the best of our knowledge, reports of HLH in SCID from developing nations are not available.

## 2 Methods

Medical records of children diagnosed with SCID at the Allergy and Immunology Unit, Advanced Pediatrics Centre, Post Graduate Institute of Medical Education and Research over the last 2 decades were retrieved and analyzed. Clinical data included demographic details, family history clinical examination findings, and pattern of infection, number of infections, type of infections, site of infections, organism involved, age of presentation, age of onset, presence of skin rash, and BCG ulceration. Hematological parameters included complete blood count, coagulation profile, serum fibrinogen levels, and bone marrow examination findings. Biochemical investigations including liver enzymes, ferritin, renal functions, lipid profile, and C-reactive protein were also analyzed.

Diagnosis of SCID was based on laboratory or genetic documentation. Diagnosis of HLH was made on the basis of HLH 2004 criteria (5). Analysis of lymphocyte subsets by flow cytometry was carried out in all patients. Laboratory assay of lymphocyte subsets, naïve, memory T cells, HLA-DR expression, CD132 expression, and lymphocyte proliferation assays were carried out as previously described (6). Immunoglobulin levels were estimated by nephelometery.

### 2.1 Lymphocyte Subset Analysis By Flow Cytometry

A total of 50 µL of EDTA blood sample is mixed with 4 μL of antibody mixture (CD45 ECD-*Beckman Coulter*), B cells (CD19 FITC- *Beckman Coulter*), T lymphocytes (CD3 PE CY7- *Beckman Coulter*), and natural killer cells (CD56 APC- *Beckman Coulter*). The mixture is vortexed and then incubated in the dark for 20-30 min at room temperature. A total of 1 ml RBC lysis buffer was then added and incubated for 15 min at room temperature. Centrifugation at 1500 rpm for 5 min was done and supernatant was decanted. There was 1 ml sheath fluid added to the pellet for washing and the tubes are centrifuged again at 1500 rpm for 5 min. The pellet was then resuspended in 300-500 μL sheath fluid. A sample was then acquired on the Beckman Coulter™ *Navios* flow cytometer. Lymphocytes were first gated using SSc vs. CD45 and different subsets were then estimated on gated lymphocytes. Analysis was done using *Kaluza* software.

### 2.2 Surface CD132 Expression By Flow Cytometry

There was 50 µL of EDTA blood sample mixed with 4 μL of antibody – CD132 PE (*Becton Dickinson*). The mixture is vortexed and then incubated at room temperature in the dark for 20-30 min. A total of 1 ml RBC lysis buffer was then added and incubated for 15 min at room temperature. Centrifugation at 1500 rpm for 5 min was done and supernatant was decanted. There was 1 ml sheath fluid added to the pellet for washing and the tubes are centrifuged again at 1500 rpm for 5 min. The pellet is now resuspended in 300-500 μL sheath fluid. A sample was then acquired on the Beckman Coulter™ *Navios* flow cytometer.

Lymphocytes, monocytes, and neutrophils were gated from the FS vs. SS plots and surface expression of common γ chain (CD132) on lymphocytes, monocytes, and neutrophils was done and compared with healthy controls. Analysis was done using *Kaluza* software.

#### 2.2.1 Molecular Analysis

Molecular analysis for patients (P2, P3) was performed at our institute. Molecular diagnosis for 2 patients (P5, P6) was established at Kazusa DNA Research Institute, Japan and National Defense Medical College, Saitama and Tokyo Medical and Dental University, Tokyo, Japan. Molecular tests for 2 patients (whole-exome sequencing) were carried out from a private laboratory in India (P1, 4). Next-generation sequencing (Ion Torrent, Thermo Fisher Scientific India Pvt. Ltd.) for clinical care was started in July 2018 at the Advanced Pediatrics Centre, PGIMER, Chandigarh. A targeted PID gene panel comprising 44 genes was used that covered 7 genes for SCID – *ADA*, *RAG1*, *RAG2*, *IL2RG*, *JAK3*, *IL7RA*, and *LIG4.* Antenatal diagnosis was performed for 3 families of these patients.

### 2.3 Search Strategy

We searched Pubmed, MEDLINE, Embase, and Scopus databases for published literature using the following search term on December, 2021: severe combined immunodeficiency and hemophagocytic lymphohistiocytosis. A total of 53 articles were reviewed and studies and case reports and series which showed development of HLH in a SCID patient were selected and reviewed (**Table 5**)

## 3 Results

Over the last 20 years, we have diagnosed 94 children with SCID at our center (7). Six children were noted to have developed HLH-like manifestations and 4 patients fulfilled the HLH-2004 criteria for diagnosis of HLH. The remaining 2 patients were considered to have a probable HLH. In these 6 patients, the male-female ratio was 5:1. Median (inter-quartile range) age of onset of clinical symptoms of SCID (onset of first documented infection) was 4.25 months (2-5 months). Median (inter-quartile range) delay in diagnosis of SCID was 1 month (1-3.5 months). Family history of deaths was seen in 4 cases. Molecular defects in *IL2RG* were seen in 5 out of 6 cases. However, final genetic diagnosis is not available in one patient (P3) as NGS for targeted PID panel performed at our center has not yielded any defect. Documented infections include disseminated bacillus calmette-guerin (BCG) infection (n=2), blood stream infections (n=3) with *Staphylococcal aureus* (n=1), *Klebsiella pneumonia* (n=1), and *Pseudomonas aeruginosa* (n=1), pneumonia (influenza H1N1 strain and K. *pneumoniae* (n=1)).

Features of HLH were noted at the time of presentation in 2 (P1, P3) children and during the hospital stay in the rest. Fever and splenomegaly were noted in 6 (100%) and 5 cases (83.3%), respectively. Laboratory features of cytopenia, hyperferritinemia, hypertriglyceridemia, and hypofibrinogenemia were seen in 6 (100%), 4 (66.6%), 2 (33.3%), and 4 (66.6%) cases, respectively (**Table 1**). While bone marrow evidence of HLH was documented in 3 cases, post-mortem histopathological evidence was seen in 2 cases.

**Table 1:** Clinical and laboratory features of HLH in patients with SCID from our cohort.

Investigations /clinical	P1	P2	P3	P4	P5	P6
Fever	Yes	Yes	Yes	Yes	Yes	Yes
Splenomegaly	Yes	Yes	No	Yes	Yes	Yes
Hemoglobin (g/L)	7.1	4.6	6.5	9.8	6.2	5
Total leucocyte count (× 10^9^ /L) (N: 4-11)	2.14 ↓	2.34 ↓	35.4 ↑	4.17	15	18.8 ↑
Differential leucocyte counts (N%/L% /M%/E%)	85/6/8/0	81/16/2/1	92/6/0/2	85/9/5/0	69/25/6/0	90/5/3/2
Platelet counts (×10^9^ /L) (N: 150-450 ×10^9^ /L)	21 ↓	4 ↓	16 ↓	150 ↓	93 ↓	14 ↓
CRP (mg/L) (N: <10)	90	204	144	78.58	NA	NA
ESR (mm/1^st^ hour) (N:0-10)	NA	2	NA	NA	NA	NA
Ferritin (ng/ml) (N:30-400)	3365 ↑	967 ↑ 1703	36016 ↑	>2000 ↑	61900 ↑	NA
Fibrinogen (g/L) (N:2.5-5)	<0.4 ↓	2.12	<0.35 ↓	0.46 ↓	1.08 ↓	NA
Triglyceride (mg/dl) (N: <100)	217 ↑	NA	172 ↑	255 ↑	>500 ↑	NA
Aspartate Transferase (IU/L) (N:15-50)	283 ↑	246 ↑	2988 ↑	133 ↑	2280 ↑	604 ↑
Alanine Transferase (IU/L) (N:12-45)	49	72 ↑	378 ↑	116 ↑	609 ↑	50
Bone marrow	NA	hemophagocytosis	NA	hemophagocytosis	hemophagocytosis	Erythrophagocytosis
Soluble CD25	NA	89.47 IU/ml (27-189 IU/ml)	NA	NA	NA	NA
HLH 2004 Criteria	Yes	Yes	No	Yes	Yes	No
HLH diagnosed on	Day 1	Day 7 HS	Day 1	Day 9	D5 HS	Post mortem/Autopsy

P, patient; N, neutrophils; L, lymphocytes; M, monocytes, E, eosinophils; HLH, hemophagocytic lymphohistiocytosis; ESR, erythrocyte sedimentation rate; CRP, C-reactive protein; HS, hospital stay, High Low. NA, Not available.

### 3.1 Patient 1

A 6-month-old boy, second born to a non-consanguineously married couple presented with high grade fever for 1 month. Fever was associated with cough and rapid breathing that was non-paroxysmal with no postural or diurnal variations for 15 days. He also developed watery loose stools associated with excessive perianal rash and excoriation. Parents also gave history of recurrent oral thrush. For these symptoms, child was treated elsewhere with intravenous antimicrobials and referred to us in view of no improvement. His elder male sibling expired at 4 months of age with pneumonia and diarrhea (**Supplementary Figure 1**). On examination, he had pallor, oral thrush, tachypnea, and tachycardia with intercostal retractions. Abdominal examination revealed splenomegaly (4 cm below left costal margin) and hepatomegaly (palpable 4 cm below right costal margin). Blood investigation showed pancytopenia with absolute lymphocyte count (ALC) 0.128 x 10^9^/L. Liver function tests showed elevated aspartate transferase (AST) 283 IU/L (N=15-40 IU/L) and alanine transferase (ALT) 49 IU/L (N=12-45 IU/L). In view of pancytopenia, persistent fever, and hepatosplenomegaly, HLH work up was sent that revealed hyperferritnemia, hypertriglyceridemia, and hypofibrinogenemia (**Table 1**). HIV serology was non-reactive. Possibility of primary HLH vs. SCID was considered. Flow cytometry was suggestive of extremely low proportions of T lymphocytes and natural killer cells (**Table 2**). Blood culture has shown growth of *K. pneumonia*. Chest x ray (CXR) showed bilateral infiltrates and thymus shadow was absent. Gastric lavage (GL) for acid-fast bacilli (AFB) staining, cartridge-based nucleic acid amplification test (CBNAAT) for *Mycobacterial tuberculosis* and smear for *Pneumocystis jirovecii* yielded negative results. Qualitative PCR for cytomegalovirus from peripheral blood was negative. The child was treated with broad spectrum antimicrobials, IV cotrimoxazole, IV Amphotericin B, and oral 4-drug antitubercular therapy (ATT). In view of HLH, intravenous immunoglobulin (IVIg) was given at 2 gm/kg. However, the pneumonia worsened requiring mechanical ventilation and he succumbed to the illness. Genetic analysis revealed *IL2RG* defect (**Supplementary Figure 1**). Antenatal diagnosis was offered for the subsequent pregnancy for parents, and the fetus was found to be unaffected.

**Table 2:** Immunological work-up of patients with SCID and HLH-like manifestations at the time of SCID diagnosis.

Immunological work up	P1	P2	P3	P4	P5	P6
Hemoglobin(g/L**)**	7.1	5.3	6.4	9.8	6.2	5
Total leucocyte count (× 10^9^ /L) (N: 4-11)	2.14 ↑	14.9 ↓	9.5	4.17	22.6 ↑	18. 8 ↑
Differential leucocyte counts (N%/L%/M%/E%)	85/6/9/0	85/14/0.9 /0.1	92/6/0/2	85/9/4/2	87/8/3/2	90/5/3/2
Platelet counts (× 10^9^ /L) (N: 150-450 ×10^9^ /L)	21 ↓	95 ↓	390	150	276	14 ↑
**Absolute lymphocyte count** (× 10^9^ /L)	0.128	2.086	0.285	0.375	1.8	0.940
**%CD3 T lymphocytes** **Absolute counts**	2.46% (49-76% 03 (1900-5900) ↓	0.61% (51-77%) 13(2500-5600) ↓	82.87% (53-84%) 236 (2500-5600) ↓	1.7% (15-77%) 6.4 (2500-5600) ↓	95.65% (49-76%) 1721 (2500-5600) ↓	Absent
**%CD 19 B lymphocytes** **Absolute counts**	87% (14-37%) 111 (610-2600)	97.82% (11-41%) 2041 (430-3000)	5.69% (6-32%) 16 (430-3000) ↓	91.57% (11-41%) 343 (430-3000)	1.78% (14-37%) 32 (430-3000) ↓	86% (14-37%) 808 (430-3000)
**%CD 56+ NK lymphocytes** **Absolute counts**	2.51% (3-15%) 3 (160-950) ↓	0.22% (03-14%) 5 (170-830) ↓	5.63% (4-18%) 16 (170-830) ↓	2.68% (3-14%) 10 (170-830) ↓	0.53% (3-15%) 10 (170-830) ↓	NA NA
**%CD3+ CD 56+ NK lymphocytes**	0.64%	0.04%	2.07%	0.05%	1.69%	NA
**%CD 45 RO+ of CD3+lymphocytes**	–	–	89.37% (control 45.92%)	81.8% (control 24.58%)	97.91%	–
**%CD 45 RA+ of CD3+lymphocytes**	–	–	12.07% (control: 54.30%)	00.16% (control: 46.29%)	0.34%	–
**%HLA DR+ on CD3+lymphocytes**	–	–	Mild increase (15.1%) as compared to healthy control (10.18%)	–	Increased (82.15%) as compared to control (49%)	–
**CD 132**	Decreased on lymphocytes (12.07%) as compared to control (40.53%)	Decreased on lymphocytes 25.23% as compared to control (83.53%)	–	–	Decreased on lymphocytes (67.8% vs 88.3%) and monocytes (59.7% vs 88.9%)	–
**IgG(g/L)** **IgA(g/L)** **IgM(g/L)**	–	IgG: <1.46 (2.40-8.80) IgM: 0.17 (0.20-10.0) IgA: 0.24 (0.10- 0.50)	–	IgG 0.232 Ig A <0.20 IgM 0.21	IgG <0.202 IgA 0.20 IgM 0.171	IgG <0. 294 IgA <0.49 IgM <0.88
**VNTR**	NA	NA	NA	NA	No maternal engraftment	NA
**Type of SCID**	IL2RG	*IL2RG*	*NA*	*IL2RG*	*1L2RG*	*IL2RG*

P, patient; N, neutrophils; L, lymphocytes; M, monocytes, E, eosinophils; VNTR, variable number of tandem repeats. NA, Not available.

### 3.2 Patient 2

**Figure 1:**
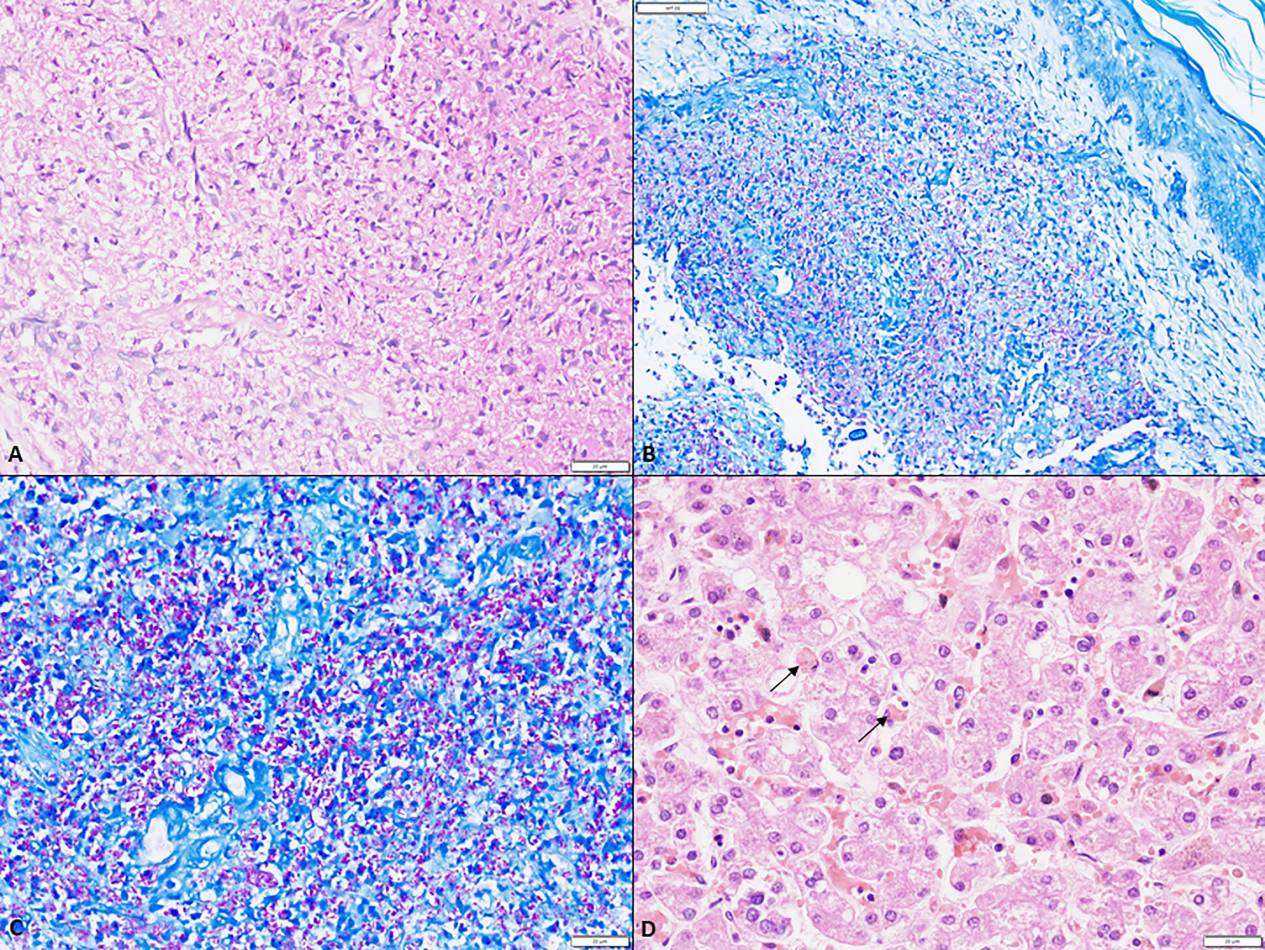
**(A)** A large collection of foamy macrophages in the dermis (scale bar 20 µm); **(B)** low magnification depicting numerous acid-fast bacilli within the foamy cells in the dermis (ZN, scale bar 50 µm); **(C)** Numerous acid fast bacilli within the dermis both intra- and extra-cellularly (ZN, scale bar 20 µm); **(D)** Hemophagocytosis within Kupffer cells in the sinusoids (arrow) (H&E, scale bar 20 µm).

A 5-month-old boy, first born to a non-consanguineously married couple, presented with high grade fever and rash for 1 month. Rash was maculopapular, non-blanchable over the entire body which healed with hyperpigmented papules. He also had cough and watery loose stools. For these symptoms, the child was treated elsewhere with intravenous antimicrobials and referred to us in view of no improvement. There was no significant family history. On examination, he had pallor, oral ulcers, palpable papules, and hyperpigmented rash all over the body. The BCG site was ulcerated with minimal pus discharge. Tachypnea and tachycardia with intercostal retractions were also noted. Abdominal examination revealed splenomegaly (9 cm below left costal margin) and hepatomegaly (palpable 5 cm below right costal margin). Blood investigation showed anemia and lymphopenia (**Table 2**). Liver function tests showed elevated liver enzymes. HIV serology was non-reactive. Possibility of SCID was considered with BCGosis in view of ulceration and pus discharge at BCG vaccination site. Flow cytometry was suggestive of extremely low proportions of T lymphocytes and natural killer cells (**Table 2**). CXR and ultrasound (USG) confirmed the absence of the thymus. USG abdomen revealed multiple tiny hypoechoic lesions in the liver and spleen. Biopsy from skin nodules showed acid-fast bacilli (**Figure 1**). Pus from the BCG site and stool have also shown AFB smear positivity, and CBNAAT positive (rifampicin sensitive) for *M.* tuberculosis complex. Hence, a diagnosis of disseminated BCGosis (lung, skin, gut, liver, spleen) with SCID was made and was started on 4-drug ATT. Infective work-up including blood cultures, and PCR for cytomegalovirus was negative. He was also given broad spectrum anti-microbials, IV Cotrimoxazole and IV Amphotericin B. In view of persistent fever despite therapy, HLH was considered. Work up was suggestive of hyperferritinemia and hypofibrinogenemia with progressive fall in platelet count and leukocyte count (**Table 1**). His soluble CD25 level was high 89.47 U/ml (27-189 U/ml). IVIg was given at 1 gm/kg. However, his pneumonia worsened, and he succumbed to the illness. Genetic analysis revealed *IL2RG* defect (**Tables 3**, **4**). Post-mortem bone marrow examination showed evidence of hemophagocytosis (**Figure 1**). Antenatal diagnosis was offered for the subsequent pregnancy for parents, and the fetus was found to be unaffected.

**Table 3:** Clinical features of patients with HLH-like features in SCID in our series.

Parameters	P1	P2	P3	P4	P5	P6
Family history	Yes	No	Yes	No	Yes	Yes
Age of onset (months)	5	4	2	5	4.5	3
Age of diagnosis (months)	6	5	3	6	6	6.5
Delay of diagnosis (months)	1	1	1	1	1.5	3.5
HLH (months)	6	5	3	6	6	6.5
BCG site	No scar	Ulcerated	No scar	Normal scar	Normal scar	No scar
History of blood transfusions	No	No	Yes	Yes	Yes	No
Rash	No	Yes (BCG)	Yes (post-transfusion)	Yes	Yes	No
Omenn/GVHD/maternal engraftment	No	No	GVHD (secondary to blood transfusion)	Omenn syndrome	Omenn syndrome	No
Infection	Pneumonia Diarrhea	Pneumonia	Pneumonia Diarrhea	Pneumonia	Pneumonia Diarrhea	Recurrent pneumonias
Organism isolated by microbiological methods	Blood culture: *Kleibsiella pneumonia*	Disseminated BCG infection	Blood culture staphylococcus aureus	H1N1; K. pneumoniae (oropharyngeal)	No organism	Disseminated BCG in thymus, lungs, lymph nodes, spleen, liver, kidney, bone marrow
Treatment regimen/Immunomodulation	IVIg 1g/kg IV antimicrobials, antifungals, ATT	IVIg 1g/kg IV antimicrobials, antifungals, ATT	IVIg 2 g/kg IV antimicrobials, antifungals	IVIg IV antimicrobials, antifungals, ATT	IVIg IV antimicrobials antifungals	IV antimicrobials IVIg 1 g/kg

**Table 4:** Detailed genetic reports of SCID patients with HLH in the present series.

Patient	Gene	Exon	Protein position	c.DNA	Inheritance pattern	Type	Novelty	Pathogenicity
P1	IL2RG	Intron 7		c.924+1 G>A	X-Linked	Splice –site (Hemizygous)	Yes	Pathogenic
P2	IL2RG	5	p.E199VfsX76	c.596_598delinsTGGATTAT	X-Linked	Hemizygous –Frameshift Indel (InsTGGATTAT delAAC)	Previously reported **(7**)	**Pathogenic**
P3	NA							
P4	IL2RG	8	p.Q322X	c.964C>T	X-Linked	Nonsense (Hemizygous)	Previously reported (7)	Pathogenic
P5	IL2RG	4	p.V152A	c.455 T>C	X-Linked	Missense (Hemizygous)	Previously reported (7)	Pathogenic
P6	IL2RG	4	p.L172R	c.515T>G	X-Linked	Missense (Hemizygous)	Previously reported (7)	Pathogenic

### 3.3 Patient 3

A 3-month-old girl, unvaccinated baby, sixth born to a non-consanguineously married couple, presented with high grade fever and rash for 1 month. She developed vesicular lesions on the trunk which progressively involved the whole body which later discharged pus. Three days prior to presentation to our institute, she developed rapid breathing and watery loose stools associated with abdominal distension. For these symptoms, the child was treated elsewhere with intravenous antimicrobials and blood transfusion was given and she developed diffuse redness of the body post transfusion and diarrhea. There was a significant family history with three elder sibling deaths (**Supplementary Figure 2**). On examination, she had pallor, anasarca, and bullous pus-filled pustular lesions and erythroderma. She had tachypnea and tachycardia with intercostal retractions and bilateral crepitations. Abdominal examination revealed hepatomegaly (liver 3 cm below right costal margin). Blood investigation done elsewhere showed anemia with absolute lymphocyte count 0.285 x 10^9^/L. When she was investigated in our institute, we noted anemia, thrombocytopenia, and elevated liver enzymes. Blood culture has shown growth of *Staphylococcal aureu*s. Qualitative PCR studies for cytomegalovirus and Epstein-Barr virus (EBV) from peripheral blood yielded negative results. A possibility of SCID with graft vs. host disease (GVHD) post transfusion was considered along with HLH. HLH work up was sent which revealed hyperferritnemia, hypertriglyceridemia, and hypofibrinogenemia (**Table 1**). Flow cytometry was suggestive of SCID (**Table 2**). IVIg was given at 2 gm/kg along with IV antimicrobials including cotrimoxazole and amphotericin B. She had further respiratory worsening, refractory shock, and succumbed to the illness. A targeted PID gene panel comprising 44 genes was used that covered 6 genes for SCID – *ADA*, *RAG1*, *RAG2*, *IL2RG*, *JAK3*, *IL7RA*, and *LIG4* and HLH genes *PRF1 and STX11* but did not yield any defect among these genes.

### 3.4 Patient 4

**Figure 2:**
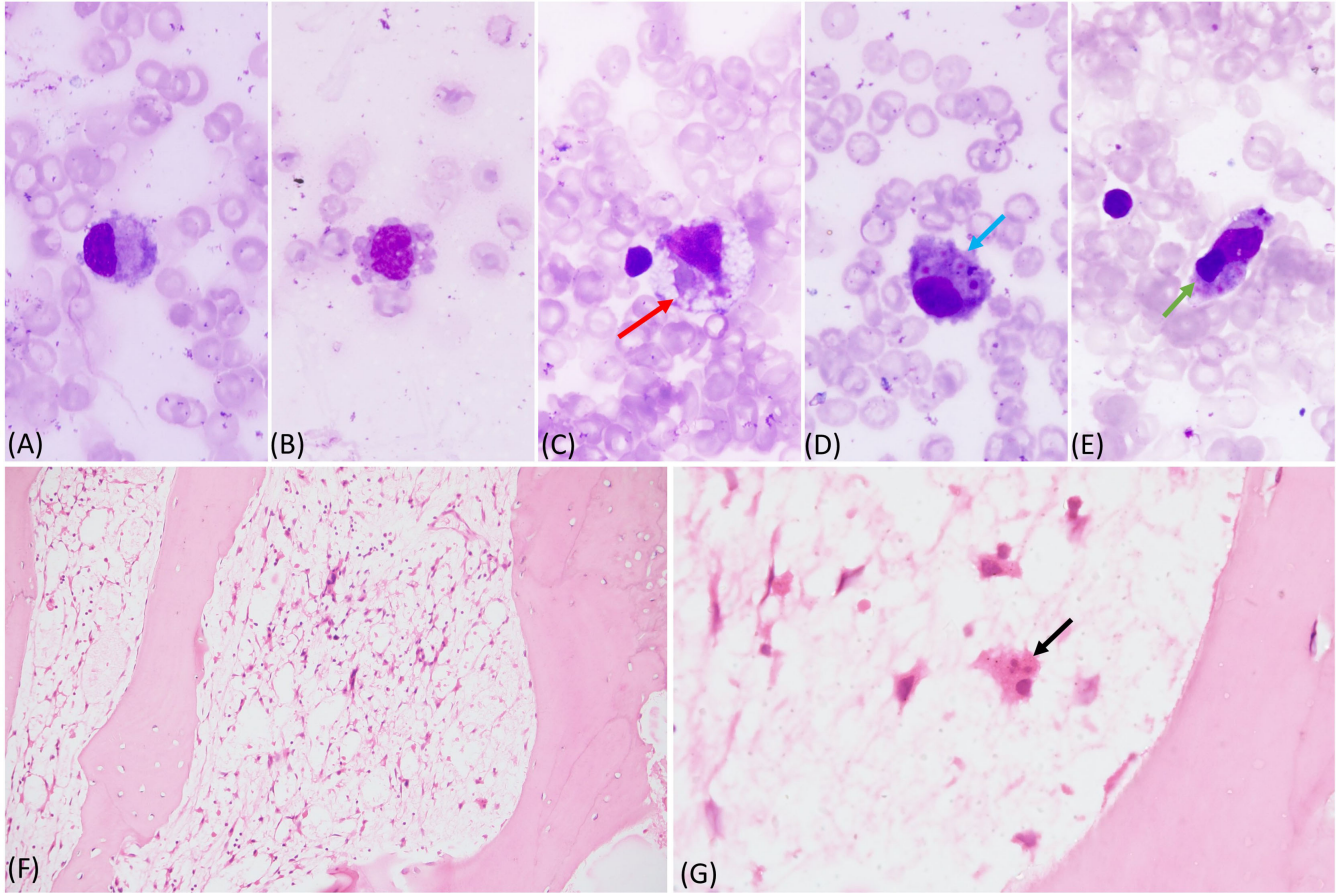
**(A-E)** Bone marrow aspirate showing histiocytes with pseudopods and vacoules, phagocytosed red cells (red arrow), platelets (blue arrow), and lymphocyte (green arrow) (May Grunwald Giemsa stain 100x); **(F, G)** markedly hypocellular bone marrow biopsy with marked reduction of all normal hematopoietic cells. A histocyte is visible with phagocytosed neutrophil (black arrow) (Hematoxylin and Eosin, F - 20x and G - 100x).

A 6-month-old boy first born to a non-consanguineously married couple presented with high grade fever and cough for 1 month. He developed rapid breathing for 15 days prior to admission. Influenza *H1N1* strain and *K. pneumonia* were isolated from nasopharyngeal secretions. He was referred to us in view of no improvement. In the past, he had watery loose stools at 1 week of age requiring hospitalization. There was no significant family history. On examination, he had pallor, tachypnea, and tachycardia with intercostal retractions and bilateral crepitations. Abdominal examination revealed splenomegaly (1 cm below left costal margin) and hepatomegaly (palpable 3 cm below right costal margin). Blood investigation showed anemia and lymphopenia. He was ventilated for progressive respiratory worsening. Work-up for *M. tuberculosis* complex and *P. jirovecii* were negative. He was treated with IV oseltamivir, and broad-spectrum antibiotics and antifungals. By Day 7 of the hospital stay, there was fall in hemoglobin, leukocyte count, and platelets, requiring blood transfusions. At this point HLH was considered and work-up revealed hyperferritnemia, hypertriglyceridemia, and hypofibrinogenemia (**Table 1**). Bone marrow was suggestive of hemophagocytosis (**Figure 2**). He was given IVIg and dexamethasone. By Day 12 of the hospital stay, he developed new onset erythematous macular rash on the cheeks that progressed to involve the whole body. A probable Omenn syndrome (OS) was considered in view of the development of lymphocytosis and eosinophilia. Liver function tests showed elevated liver enzymes. Flow cytometry was suggestive of extremely low proportions of T lymphocytes and natural killer cells (**Table 2**). Qualitative PCR for cytomegalovirus from peripheral blood was negative. Clinical symptoms further worsened, and he succumbed to illness. Genetic analysis revealed *IL2RG* defect (**Table 4**).

### 3.5 Patient 5

A 6-month-old boy developmentally normal, immunized for age, first born to a non-consanguineously married couple presented with high-grade fever and cough for 1.5 months. Cough was progressively increasing in severity and was associated with rapid breathing for 15 days prior to presentation. He also developed watery diarrhea for 15 days. For these symptoms, the child was treated elsewhere with intravenous antimicrobials. He also received a blood transfusion because of anemia. In view of worsening respiratory distress, he was referred to our center. There was a significant family history with deaths of 5 maternal uncles by the age of 6 months due to respiratory illnesses (**Supplementary Figure 1**). On examination, he had pallor, generalized macular rash all over the body, and a healthy BCG scar. Chest examination revealed bilateral crepitations. Abdominal examination revealed splenomegaly (4 cm below left costal margin) and hepatomegaly (palpable 5 cm below right costal margin). Blood investigations showed lymphopenia (absolute lymphocyte count 1.808 x 10^9^/L) and elevated liver enzymes. CXR and computed tomography of chest revealed diffuse bilateral consolidations. The child was initiated on broad spectrum IV antimicrobials. HIV serology was non-reactive. A possibility of SCID was considered with the presence of family history and lymphopenia. Flow cytometry showed low T cells, decreased naïve T cells, expanded HLA-DR, and decreased CD132 expression (**Table 2**). By Day 5 of the hospital stay, HLH was considered in view of persistent fever, transaminases, anemia, and fall in platelet count to 93x10^9^/L and work-up revealed hyperferritnemia, hypertriglyceridemia, and hypofibrinogenemia (**Table 1**). Infective work-up including blood cultures and PCR for cytomegalovirus was negative. A variable number of tandem repeats (VNTR) analysis was done and there was no evidence of maternal engraftment. In view of HLH, IVIg was given at 1 gm/kg and was planned for steroids but the child succumbed to the illness. Genetic analysis revealed *IL2RG* defect (**Table 4**).

### 3.6 Patient 6

A 6.5-month-old boy, sixth born to a non-consanguineously married couple presented with cough for 2 months. Cough progressively increased in severity and was associated with rapid breathing and fever for 15 days prior to presentation. Parents also noted abnormal body movements of left limbs with altered sensorium. The child had significant history in the form of recurrent pneumonia since the age of 3 months requiring hospitalizations. There was significant family history with the deaths of 3 male elder siblings at the ages of 2, 5, and 6 months, respectively (**Supplementary Figure 1**). On examination, there was failure to thrive and pallor. Chest examination revealed intercostal retractions and bilateral crepitations. Abdominal examination revealed splenomegaly (4 cm below left costal margin) and hepatomegaly (palpable 5 cm below right costal margin). Blood counts showed progressive pancytopenia. Blood culture showed growth *Pseudomonas aeruginosa*. CXR showed diffuse perihilar infiltrates. Work-up for cytomegalovirus and EBV was negative. A possibility of SCID was considered in the presence of family history and lymphopenia. Flow cytometry showed absent T lymphocytes (**Table 2**). He was treated with antimicrobials and IVIg 1 gm/kg for SCID, however, he succumbed to illness by Day 9 of stay. An autopsy was performed that showed thymic atrophy, marked lymphoid depletion, disseminated *BCG* in the thymus, lungs, lymph nodes, spleen, liver, kidney, and bone marrow, and bronchopulmonary aspergillosis and erythrophagocytosis in bone marrow and lymph nodes. Genetic analysis revealed *IL2RG* defect (**Table 4**). Antenatal diagnosis was offered for the subsequent pregnancy for parents, and the fetus was found to be unaffected.

## 4 Discussion

Ours is the first study of HLH-like manifestations in children with SCID from the Asia Pacific region. The frequency of this rare, yet life-threatening manifestation in our series is 6.38% (6/94) with pathogenic mutations in *IL2RG* in 5 out of 6 cases. Infective triggers have been documented in 5 cases and GVHD/Omenn phenotype was noted in 3 cases.

The first case report of HLH in SCID was reported in 2000 by Grunebaum et al. (8) in a 9-week-old child with X-linked SCID. This was a presenting manifestation of SCID and there were no infections identified at onset. Subsequently, several other case reports have been published (9–18) (**Table 5**). Later, Bode et al. (3) described a larger cohort of cases (n=63) with PID who developed HLH. In this study, 12 patients had SCID and 18 had partial T-cell deficiencies. The most common mutation in these SCID was that of *IL2RG* (n=5), followed by *RAG1* (n=2). In another study by Cetinkaya et al., 4 had SCID, of which mutations in the *RAG1* were identified in 2 patients (4). With the available literature, the most common types of SCID to develop HLH or HLH-like manifestations are X-linked SCID followed by RAG defects. However, features of HLH have also been described with *JAK3*, *CD3D*, *ADA*, and *ORAI1* defects also (13) (15) (16) (18), (20). In India, autosomal forms of SCID are more common than X-linked SCID. However, we observed that the most common type of SCID associated with HLH was *IL2RG* defect (X-linked SCID). Hence HLH in an infant, especially less than 6 months with an X-linked family history should guide us to investigate for SCID, as almost all primary HLH that have been described to date are autosomal recessive in nature. Increased incidence of HLH in *IL2RG* defect is probably due to the defective natural killer function. We document a wide range of mutations – missense, splice-site, and frameshift defects in *IL2RG* in our patients who have developed HLH. Therefore, it appears that type of mutation has no influence on the development of HLH in X-linked SCID.

**Table 5:** Review of literature of previously reported cases of HLH in SCID patients.

Author/Ref.	Genetic	Age (median age (IQR)/Sex	Infections	HLH onset age	HLH features	Management of HLH	HSCTY/N	Outcome
Chidambaram et al., 2020India (16)	Homozygous missense variation in exon 11 of the ADA gene	3 month/F	CMV PCR positivity (bloodand urine)	3 months	Anemia, thrombocytopenia; high ferritin (13797 ng/dL); high serum triglycerides (532 mg/d)	IVIgDexamethasone; Ganciclovir	No	Expired
Bode et al., 2015 (3)	12 patients IL2RG:5 RAG 1:2 IL7RA:1 CD3E:1 Unidentified: 3	NA	CMV (n = 3), adenovirus (n = 3), EBV (2), M TB (n = 1), *Enterobacter* sp. (n = 1), gram-negative (n = 1), *P.* aeruginosa (n = 1) rhinovirus (n = 1), *Pneumocystis jirvoceii* (n = 1)	0.13-1.5 years	–	Corticosteroids alone or in combination with intravenous immunoglobulins, cyclosporine, or etoposide	NA	8 Expired 4 Survived
Cetinkaya, Turkey 2020 (4)	4 patients 2 RAG	2.5 months (2-5 months) M:F:3:1	CMV (n = 3) and parainfluenza 3 (n = 1)	4.5 months (2-22 months)	–	Corticosteroids alone or in combination with intravenous immunoglobulins, cyclosporine, or etoposide	Y (N = 1)	Expired 3 Alive:1 (HSCT)
Shi et al., 2020; China (17)	IL2RG gene (Exon 6: c.854G > A; p.Arg285Gln)	4 month/M	*M. tuberculosis* *M. bovis*	4 months (Day 8 of HS)	Low fibrinogen (0.91 g/L); high ferritin 3235 ng/mL; high soluble CD25 cells (5182.51 pg/mL)	One intravenous etoposide (40 mg, IV in one dose) Dexamethasone (2 mg IV every 12 h)	No	Expired
Patirgolu et al., 2014 (14)	IL2RG gene; the novel mutation in exon 5 (c.595-1G>T)	3 month/M	*Candida albicans* (blood culture) *Pseudomonas aeruginosa* (aspirated tracheal fluid)	3 month, (3rd week of HS)	Elevated transaminases, pancytopenia, high triglycerides and ferritin, low fibrinogen, hemophagocytic histiocytes in bone marrow	IVIG, broad-spectrum antibiotics, ATT	No	Expired
Grunebaum et al., 2000 (8)	IL2RG SCID	9 week/M	NA	9 weeks (at onset)	Pancytopenia ; high triglycerides and ferritin, low fibrinogen; bone marrow of histiocytes with hemophagocytes	Etoposide and dexamethasone	No	Expired; Gram-negative septicemia
Alsalamah., 2015 (15)	Homozygous mutation in the CD3δ gene	6 month/F	Adenovirus (nasopharyngeal swab); urine culture *Klebsiella pneumoniae*	6 months (at onset)	Anemia (Hb 88 g/L), thrombocytopenia; leukopenia; high ferritin (>100,000 μg/L), elevated triglyceride (12.24 mmol/L); hypofibrinogenemia (1.37 g/L) and high soluble IL-2 receptor (4683 U/mL)	Dexamethasone, IVIG, corticosteroids, and etoposide	No	Expired 2 weeks (ongoing HLH, refractory bleeding, and encephalopathy)
Suzuki et al., 2009 Japan (11)	NA	19 d/F	–		5/8 HLH	IVIG, corticosteroid, cyclosporine, etoposide, HSCT	Y	Expired
Schimid et al. (9)	(T−, B+, NK+) SCID Genetics NA	5 month/M	Active EBV infection was diagnosed by quantitative PCR testing (675,000 genome equivalents/20,000 cells).	6 days of HS	Anemia, thrombocytopeniaand leukocytosis; ferritin (5866 ng/ml) and triglycerides (241 mg/dl), erythrophagocytosis	Dexamethasone Cyclophosphamide Low dose Etoposide IVIG	Y	Expired (ARDS Aspergillus in ET)
Dvorak et al., 2008 (10)	X-linked SCID	7 week/M	Methicillin-sensitive *Staphylococcus aureus*At 7 weeks: Rhinovirus (nasopharyngeal swab); positive for *Enterobacter aerogenes* (blood culture)	7 weeks of life	Bone marrow active hemophagocytosis, elevated serum levels of ferritin (872 ng/mL; normal <500 ng/mL), and soluble interleukin-2 receptor a chain (CD25) (9016 pg/mL), normal (239 to 7887 pg/mL),_ severe anemia, thrombocytopenia was moderate with a nadir of 36,109/L platelets	IVIG, 2-week course of cyclosporine (3 mg/kg/dose twice a day, adjusted for a goal level of 250 to 300 ng/mL): no response	Y	Well at 17 months
Klemann et al., 2017 (20)	ORAI1	6 weeks/M	CMV infection was diagnosed based on of blood virus loads >100.000 IU/ml *Pneumocystis jirovecii* pneumonia	3 months of age at presentation	Pancytopenia; hyperferritinemia (5103ng/ml), hypertriglyceridemia (371 mg/dl = 4.1 mmol/l), hypofibrinogenemia (1.4 g/l) and elevated soluble CD25 (max. 4022 U/l).	Treatment with dexamethasone improved the HLH symptoms, but the patient relapsed upon tapering	Y	Expired (severe, CMV-associated pulmonary inflammatory complications)
Norris et al., 2009 (12)	IL2RG	5 month/M	*Pneumocystis jirovecii.* Pneumonia. and parainfluenza virus type 3 The posttransplant course was complicated by numerous infections including persistent parainfluenza, *Corynebacterium* sp., *Enterococcus faecalis*, *Staphylococcus epidermis*, *Staphylococcus hominus*, *Staphylococcus haemolyticus*, *Serratia* *marcescens*, and *Clostridium difficile* colitis.	Post-HSCT	Pancytopenia; extensive lymphohistiocytic infiltrate with evidence of mild hemophagocytosis	4 weekly doses of rituximab (375 mg/m2) in addition to his immunosuppression with tacrolimus and prednisolone	Y	Chimerism continues to be 85% donor 20 months from second HSCT, and immunologic reconstitution is normal
Tucci, 2021 Italy (18)	ADA SCID	4 year/F	*Mycobacterium bovis* *Stenotrophomonas maltophilia* bacteremia, invasive pulmonary aspergillosis, adenovirus reactivation	Post 2nd HSCT (D+13)	Persistent fever, hepatosplenomegaly, high levels of triglycerides (383 mg/dL) and markedly elevated ferritin (18,000 mg/dL) and soluble IL2 receptor (16,809 pg/mL; BM morphology showed active hemophagocytosis	Methylprednisolone (2 mg/kg/day); high-dose immunoglobulins; Emapalumab max 6 mg/kg; surgical incision of the abscesses; anti-TB treatment	Y; 3 HSCT	Alive after 3rd HSCT Full donor chimerism at Day +100 post HSCT
Hashi et al., 2010 China (13)	Novel homozygous non-sense mutation of JAK3 (C623T; R175X)	5 month/f	NA	Post HSCT (Day 18)	Cytopenia; BM aspiration revealed hypoplastic marrow with hemophagocytosis; high serum ferritin (715 ng/mL) and serum soluble IL-2 receptor level (3295 U/mL)	Etoposide 30 mg/m^2^ and pulse methylprednisolone (30 mg/kg)	Y	Expired Day 32 post-HSCT (respiratory failure)
Singh et al., 2020 USA(19)	IL2RG	10 day/M	Human herpes virus 6	10 days of life	Fever and pancytopenia with elevated ferritin (1251 ng/mL) and LDH (457 IU/mL) levels	Dexamethasone	Y	Alive

NA, Not available

Most of the time, HLH in PIDs/SCID is triggered by infections. Attempts to isolate an organism becomes important in the management of HLH. Management of secondary HLH can be challenging especially in cases of SCID because the presence of severe infections may hinder the use of aggressive immunosuppression. In the series by Bode et al. (3), 50/63 PID patients (79%) with HLH syndrome had associated infections. In 12 children with SCID, the most common organisms isolated were cytomegalovirus CMV (n=3), adenovirus (n=3), EBV (n=2), *M. tuberculosis* (n=1), Enterobacter sp. (n=1), gram negative (n=1), *P. aeruginosa* (n=1), rhinovirus (n=1), and *P. jirovecii* (n=1). Also, in the study by Cetinkaya et al., CMV (n=3) and parainfluenza 3(n=1) (4) were documented. Features of HLH were mostly associated with viral infections. However, the infections were most commonly bacterial in our cohort with 2 cases of disseminated BCGosis. Viral infection was identified in one patient only (17%) (P4). The increased risk of BCGosis in our setting is due to the lack of universal screening of SCID and effective universal vaccination with BCG vaccine to all newborns on Day 1 of life. However, the co-infections with viruses cannot be excluded due to lack of availability of molecular tests for viruses in our setting. Hence, in a setting of HLH with life threatening proven infections, either bacterial or viral, PIDs such as SCID need to be important differential diagnoses.

Most of the children with SCID have isolated lymphopenia at diagnosis and ALC in hemogram gives a clue to make a diagnosis. However, lymphopenia can also occur as a part of pancytopenia in HLH. In such cases, disproportionate reduction in T cell proportions, decrease in naïve T cells, altered CD4/CD8 ratio, and decreased lymphocyte proliferation provide laboratory clues toward underlying SCID (6). In our series, lymphopenia was seen in all patients; however, pancytopenia/bicytopenia (P1, P2, P6) was noted in 3 out of 6 cases. In these patients, an extremely low proportion of T cell percentage and decrease in naïve T cells provided vital clues toward underlying SCID.

Bode et al. (3) showed lower levels of serum soluble interleukin-2 receptor (sCD25) and higher ferritin levels in HLH associated with T-cell deficiencies compared to HLH in other PIDs. The authors also proposed that the ratio of ferritin:sCD25 ≥3 as a clue to suspect SCID/CID in a child with HLH. We could perform sCD25 levels in one patient and the levels were normal (P2). In this case, the ratio of ferritin and sCD25 was also high (19.3).

Usually, it is the activated T lymphocytes that are involved in immunopathogenesis of HLH (21) (22). CD8+ T cell activation leads to interferon overproduction and macrophage activation. In patients with SCID and combined immunodeficiency, HLH-like manifestations occur despite severe T-cell deficiency/impairment (3) (8). Lack of regulation of excess immune response by T cells due to defective IL-2/IL-2R system could possibly explain development of hyper-inflammatory complications in SCID (23).

Engrafted maternal T cells with oligoclonal expansion survive for a long duration in SCID (24). These activated maternal T cells can result in HLH (25) (26) (27). Dvorak et al. (10) showed that maternal CD8 T cell engraftment was a key driver for HLH. Similarly, HLH as a result of donor T-cell engraftment has also been shown to occur in children with SCID with post-HSCT (12) (13). Hence, host macrophage activation was presumably induced in response to donor/maternal engrafted T lymphocytes through immunoreaction to infections and/or alloantigens. The presence of these T cells is the likely source of the elevated circulating CD25 levels in such cases.

In simple terms, donor lymphocytes respond to host cells or resident infectious organisms, leading to IFN production and activation of host macrophages. In our series too, 3 children had GVHD/Omen like phenotype (P3,4,5). VNTR was performed in one child (P5) and maternal engraftment was ruled out.

Another pathogenesis for HLH is the activation of innate immunity. Gain-of-function mutations in NLRC4, a protein that activates an inflammasome, have been documented in cases of recurrent MAS (28–31). Further studies on innate immunity in cases of SCID with HLH-like manifestation can throw light into these new pathways.

Management of HLH-like manifestations in SCID involves identification of the infective trigger, aggressive management of infections, and early hematopoietic stem cell transplantation (HSCT). However, optimal immunomodulatory strategies for management of HLH in SCID is still not clear. Supportive therapy with IVIg and immunomodulatory therapies for HLH were used for management with hardly any success. Bode et al. (3) reported usage of IVIg in 4 patients, and steroid, etoposide, or HLH 1994/2004 (5, 32) protocol in 5 patients. Cetinkaya (4) reported 3/4 SCID patients (75%) died of HLH before HSCT and treatment with IVIg, dexamethasone, cyclosporine, and etoposide were tried. IVIg was used as a first line for managing of HLH in our series with no success. In our setting, none of the patients were able to reach the process of HSCT due to the serious illness and infections owing to delayed diagnosis. HLH can still occur post-HSCT, probably due to engraftment of donor cells and concurrent infections (12, 13) (18). Recently, emapalumab (18) has been successfully used in a child with recurrent HLH in SCID who underwent HSCT.

When short of HSCT, SCID is fatal. In the series by Bode et al. (3), 8/12 children with SCID HLH died, which is much higher than that of CGD (2/22). However, data on HSCT are not available. Cetinkaya (4) reported 3 of 4 SCID patients (75%) died of HLH before HSCT. In our study, all died due to delayed presentation and diagnosis, which probably must have led to fulminant uncontrolled infections and life threatening HLH. This again calls for the need of increasing awareness of SCID and its varied HLH-like presentation.

Various types of infections including viral, bacterial, and parasites have been shown to trigger HLH (33–36), However the exact mechanism of infections triggering HLH is unclear and the margin to differentiate HLH and infections causing sepsis is blurred. The probable mechanism of susceptibility to HLH could be uncontrolled infection with high antigen load resulting in cytokine storm and inhibiting apoptotic pathways. Also, a direct connection between the viral infection and inhibition of natural killer cell or T cell cytotoxicity was documented (35).

In children with SCID who developed HLH, there is also a possibility of the presence of concomitant genetic defects in any one of the genes associated with congenital HLH. However, NGS performed in our patients did not yield any variants in *PRF1* or *STX11* genes in 2 of them. Two patients who underwent a whole exome sequencing in a private laboratory did not reveal any pathogenic variants in the genes implicated for congenital HLH. Moreover, autopsy performed in 2 patients did reveal classical features of SCID such as thymic atrophy, lymphoid hypoplasia, and opportunistic infections apart from HLH. This suggests that the etiology of HLH-like manifestations in patients with SCID is likely acquired or secondary to infection. However, we have performed a whole exome in only 2 of our patients and, therefore, we cannot conclusively state that HLH in patients with SCID is only acquired and not congenital in origin.

## 5 Conclusion

HLH-like manifestations secondary to infections can be the presenting features of PID and diagnosis of SCID in such situations can be challenging. In such a setting, the presence of a suggestive family history, associated infections, and disproportionate T cell reduction in flow cytometry in such settings provide clues to underlying SCID. Mortality is high in infants with SCID who had HLH-like manifestations and the role of immunomodulatory therapy in these cases is not clear. Establishment of genetic diagnosis can help in antenatal diagnosis in future pregnancies of the affected families.

## Data Availability Statement

The original contributions presented in the study are included in the article/**Supplementary Material**. Further inquiries can be directed to the corresponding authors.

## Ethics Statement

Ethical review and approval was not required for the study on human participants in accordance with the local legislation and institutional requirements. Written informed consent to participate in this study was provided by the participants’ legal guardian/next of kin. Written informed consent was obtained from the individual(s) for the publication of any potentially identifiable images or data included in this article.

## Author Contributions

PV – Inception of idea, editing of the draft, clinical management, intellectual input, and final approval of the manuscript. GA – Preparation of the draft, clinical management, review of literature, final approval. RK – Preparation of the draft, laboratory work-up, final approval.

AS, SM, and JN – Clinical management, review of literature, final approval. AJ and DS – Clinical management, editing of the draft, final approval. MS, GK, and SSh– laboratory work-up, final approval. KG and SSr – laboratory work-up, editing of the draft, final approval. AR – editing of the draft, laboratory work-up, intellectual input, and final approval of the manuscript. SSi – editing of the draft, final approval. All authors contributed to the article and approved the submitted version.

## Conflict of Interest

The authors declare that the research was conducted in the absence of any commercial or financial relationships that could be construed as a potential conflict of interest.

## Publisher’s Note

All claims expressed in this article are solely those of the authors and do not necessarily represent those of their affiliated organizations, or those of the publisher, the editors and the reviewers. Any product that may be evaluated in this article, or claim that may be made by its manufacturer, is not guaranteed or endorsed by the publisher.

## Supplementary Material

Supplementary Figure 1 **(A)** Decreased surface expression of CD132 on lymphocytes in patient 1 (12.07%) as compared to control (40.53%). **(B)** Sanger plots of patient 1 and mother showing hemizygous state of mutation in the patient and heterozygous carrier state in the mother.

Supplementary Figure 2 Family pedigree charts of patients with SCID and HLH-like manifestations (P1, P3, P5, P6).
