# Supplementary figures and images for: Features of Hemophagocytic Lymphohistiocytosis in Infants With Severe Combined Immunodeficiency: Our Experience From Chandigarh, North India
Source: Front Immunol. 2022 Jun 23;13:867753. doi: 10.3389/fimmu.2022.867753 (PMC9260510; doi:10.3389/fimmu.2022.867753)

## Control

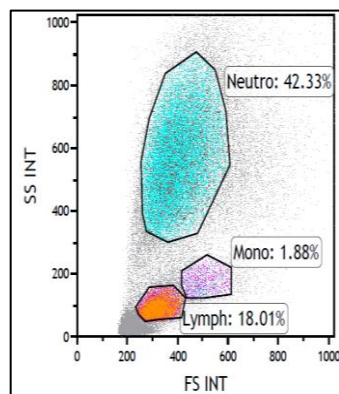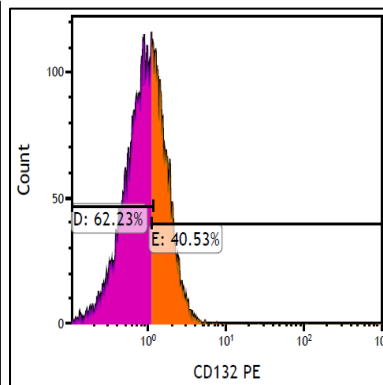

CD132 on lymph

## Patient

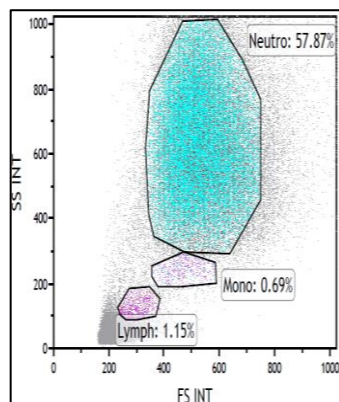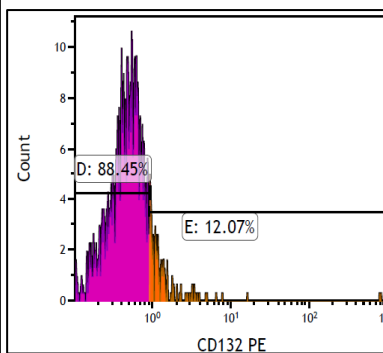

CD132 on lymph

Supplement: Supplementary Figure 1 — (A) Decreased surface expression of CD132 on lymphocytes in patient 1 (12.07%) as compared to control (40.53%). (B) Sanger plots of patient 1 and mother showing hemizygous state of mutation in the patient and heterozygous carrier state in the mother. [file DataSheet_1.pdf]
