# Supplementary material for: Features of Hemophagocytic Lymphohistiocytosis in Infants With Severe Combined Immunodeficiency: Our Experience From Chandigarh, North India
Source: Front Immunol. 2022 Jun 23;13:867753. doi: 10.3389/fimmu.2022.867753 (PMC9260510; doi:10.3389/fimmu.2022.867753)
Supplement: Supplementary Figure 2 — Family pedigree charts of patients with SCID and HLH-like manifestations (P1, P3, P5, P6). [file DataSheet_2.pdf]

Sanger validation for Patient 1
c.924+1G>A (Splice-site variant)

Patient 1- affected (a&b)

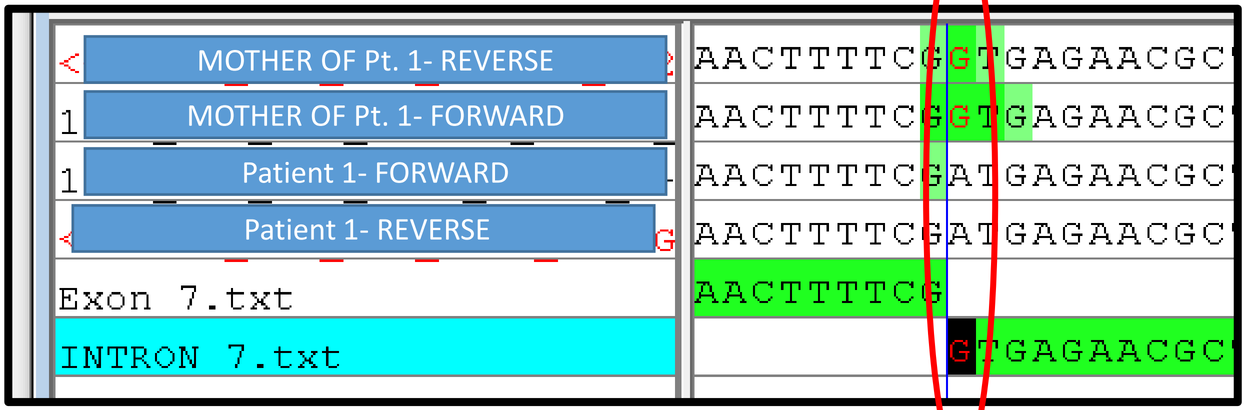

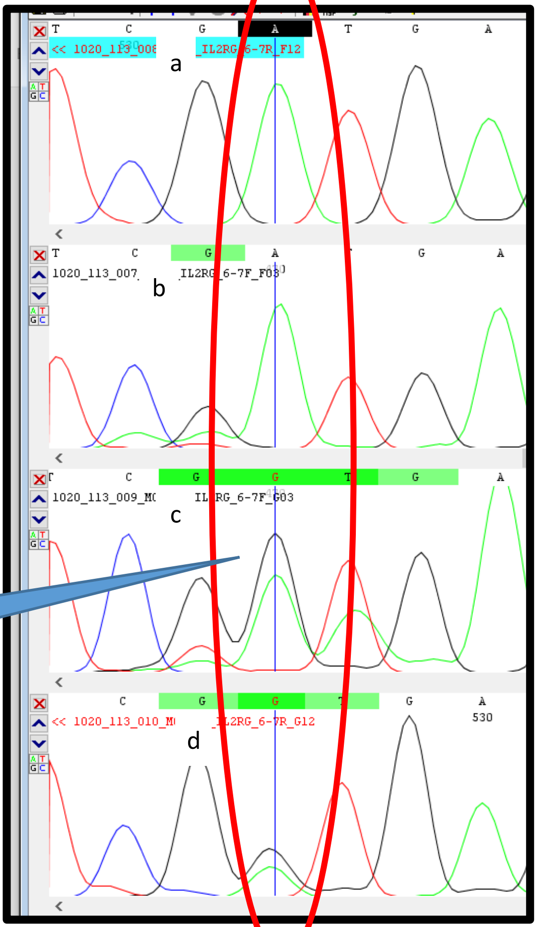

Mother is carrier (c &d)
